# Supplementary material for: Assessment of carbon monoxide in exhaled breath using smokerlyzer among in-school adolescents in Osun State: A cross sectional study
Source: PLoS One. 2026 Apr 28;21(4):e0347739. doi: 10.1371/journal.pone.0347739 (PMC13123932; doi:10.1371/journal.pone.0347739)
Supplement: Attachment — Submitted filename: Reviewing points.docx [file pone.0347739.s001.docx]

Reviewing points:

1- The introduction should be revised and rewritten.

2- The introduction does not mention the possible risks and side effects of smoking in adolescents.

3- The study objectives are not well stated at the end of the introduction.

4- The data collection method needs to be revised and explained further.

5- In performing data analysis, in addition to logistic regression, a rank regression analysis should also be considered. Because the response variable of smoking is defined as a rank variable.

6- The entire text of the article should be shortened.

7- Grammatical and reference errors should be corrected.
