# Supplementary material for: Assessment of carbon monoxide in exhaled breath using smokerlyzer among in-school adolescents in Osun State: A cross sectional study
Source: PLoS One. 2026 Apr 28;21(4):e0347739. doi: 10.1371/journal.pone.0347739 (PMC13123932; doi:10.1371/journal.pone.0347739)
Supplement: Attachment — Submitted filename: Responses_reviwers_comments_PLOSONE_25.docx [file pone.0347739.s002.docx]

RESPONSE TO THE REVIEWERS COMMENTS COMMENTS RESPONSES

**Reviewer 1**

| S/N | COMMENTS | REPONSES | Page |
| --- | --- | --- | --- |
| 1 | The rationale for testing only 50% of participants with the Smokerlyzer should be clearly explained. Was this due to budget constraints, time limitations, or another methodological consideration? | This was due to financial constraints | Page 5  Lines 102-103 |
| 2 | Several adjusted odds ratios reported in the logistic regression (e.g., age group and sex) have confidence intervals that appear excessively wide or imprecise. Please review these values to confirm their accuracy. The interpretation of Cohen’s Kappa (κ = 0.048) as “purely coincidental” should be revised to reflect standard interpretations (i.e., "slight agreement"). | The values have been reviewed | Page 16-17  Lines 264-265  Page 13-14  Lines 230-232 |
| 3 | The study reports associations between school environment (lack of health clubs, absence of anti-smoking materials) and smoking behavior. While these are interesting findings, potential residual confounding (e.g., school-level SES, teacher influence, peer smoking norms) should be acknowledged and discussed as a limitation. | done | Lines 342-345  Pages 21-22 |

**Reviewer 2**

| S/N | COMMENTS | REPONSES | Page |
| --- | --- | --- | --- |
| 1 | The introduction should be revised and rewritten. | Introduction has been revised and rewritten | Pages 3-4  Lines 43-76 |
| 2 | The introduction does not mention the possible risks and side effects of smoking in adolescents. | These have been included | Pages 3-4  Lines 56-65 |
| 3. | The study objectives are not well stated at the end of the introduction. | They have been included | Page 4  Line 75-76 |
| 4. | The data collection method needs to be revised and explained further. | This section has been revised and further explained | Page 7  Lines 134-151 |
| 5. | In performing data analysis, in addition to logistic regression, a rank regression analysis should also be considered. Because the response variable of smoking is defined as a rank variable | A table on rank regression analysis has been included | Page 25  Lines 280-281 |
| 6. | The entire text of the article should be shortened. | Done |  |
| 7. | Grammatical and reference errors should be corrected. | Done |  |
| 8. | Additional References have been included while the introduction was rewritten. |  | Lines  365-440  Pages22-25 |
