# Supplementary material for: Assessment of carbon monoxide in exhaled breath using smokerlyzer among in-school adolescents in Osun State: A cross sectional study
Source: PLoS One. 2026 Apr 28;21(4):e0347739. doi: 10.1371/journal.pone.0347739 (PMC13123932; doi:10.1371/journal.pone.0347739)
Supplement: Attachment — Submitted filename: Response_to_reviewer2026_PLOSone.docx [file pone.0347739.s003.docx]

RESPONSE TO THE REVIEWERS COMMENTS

Manuscript Title: Assessment of Carbon Monoxide in Exhaled Breath using Smokerlyze among in-school adolescents in Osun State: A Cross Sectional Study

**Reviewer 1**

| S/N | COMMENTS | Action required | Responses | Page |
| --- | --- | --- | --- | --- |
| 1 | Statistical Inconsistency & Multicollinearity (Page 21-22, Tables 7 & 8) I note that in response to previous reviewer comments, you have included a rank regression analysis (Table 8) alongside the logistic regression (Table 7). However, the results of these two models are contradictory regarding the key predictors: age and class. In the logistic regression (Table 7), age is a risk factor (AOR=1.59), while class is a protective factor (higher classes have lower odds). In the Rank Regression (Table 8), age appears protective (negative coefficient), while class is a strong risk factor (positive association). | Please check the Variance Inflation Factor (VIF) for age and class in your models. If high multicollinearity exists, you may need to remove one of these variables or combine them to ensure the reported predictors are accurate. You must explain or resolve this contradiction in the results; currently, the conclusion that "older age and class are predictors" is ambiguous because the direction of the effect depends on which table the reader looks at | Thank you for this important observation. We would like to clarify that the apparent difference in direction of association between Tables 7 and 8, arises from differences in outcome definition and model interpretation, rather than statistical inconsistency or multicollinearity.  Table 7 presents a binary logistic regression modelling the likelihood of being a smoker versus a non-smoker, whereas Table 8 applies rank regression to model the relative ordering of smoking behaviour. Rank regression does not estimate risk or severity but rather examines associations with higher or lower ranks of the outcome.  Accordingly, coefficients in Table 8 is not interpreted as indicators of smoking severity or initiation and are not directly comparable with odds ratios from logistic regression.  We have revised the table title, footnote, and manuscript text (Pages 21–22) to explicitly clarify this distinction and to prevent misinterpretation.  In addition, diagnostic checks showed no evidence of problematic multicollinearity among predictors (all VIFs < 2). | Pages 17 -18  Lines 264-275 |
| 2 | Selection Bias in Sub-sample (Methodology Section) You state that due to financial constraints, 50% of the survey sample was selected for the Smokerlyzer assessment (n=616). | Action Required: Please explicitly clarify the randomization method used to select this 50\% sub-sample. If it was simple random sampling or systematic random sampling within the schools, please state this clearly to ensure the technical soundness of the 27.3% prevalence rate. | Done | Page 5  Lines 90-97 |
| 3 | Title Correction There is a typo in the manuscript title. Current: "...using Smokerlyze" Correction: Please change to "...using Smokerlyzer..." | Correction: Please change to "...using Smokerlyzer..." | Done | Line 1  Page 1 |
| 4. | 4. Discussion of Age/Class Dynamic In the Discussion section, you recommend targeted education for "older adolescents" and "lower classes." Comment: Please briefly elaborate on the interaction between these two demographics. Are the "high risk" students specifically older adolescents who have been held back in lower classes? Clarifying this profile would make your public health recommendations more actionable. |  | No,it does not and all VIFs are less than or equal to 2.This does not reveal there are interaction.  It has been corrected | Page 2  Line 38 |
| 5. | Table Formatting Please ensure the table footnotes for Tables 7 and 8 clearly define the reference categories used for the regression analyses to assist reader interpretation. |  | The reference categories for Table 7 is indicated as Ref in the table.  Table 8 is a rank regression where all variables are entered as continuous variable and not binary outcome variable, hence no reference categories are presented. | Page 17  Line 261 |

Reviewer 2

| S/N | Comments | Action required | Responses | Page |
| --- | --- | --- | --- | --- |
| 1 | In the method section, all subsections from the study site to the exclusion criteria should be combined and written in one subsection. |  | This has been done | Page 4-5  Lines 79-88 |
| 2 | In the sample size determination subsection, the relevant formula is incorrect, correct it. |  | We thank you for this observation. This study was part of a multiphase study where the initial sample size was determined using the formula (Zα + Zβ)² P (1 – P) d² . The estimate of true proportion in the population; was taken as the prevalence of media health literacy among secondary school students in Osun State 37.7%.Hence the formula that was used. A subset of the sample size (50%) was used for the objective measurement of smoking due to the financial constraints | Page 5  Lines 90-97 |
| 3. | In the final calculation of the sample size, a reference should be mentioned for applying the coefficient of 1.5 based on the category |  | This has been done | Page 5  Lines 93-94 |
| 4. | In the abstract section of the article, there is no mentioned or reported result of the rank regression. The result of the rank regression should be reported or deleted altogether. |  | This has been included | Page 2  Line 33-34 |
| 5. | A reference should be introduced for the use of rank regression. |  | This has been done | Page 8  Line 160 and 161 |
